# Transgenic reporter analysis of ChIP-Seq-defined enhancers identifies novel target genes for the terminal selector UNC-3/Collier/Ebf

**DOI:** 10.17912/micropub.biology.000453

**Published:** 2021-09-14

**Authors:** Yinan Li, Paschalis Kratsios

**Affiliations:** 1 Department of Neurobiology, University of Chicago, Chicago, IL, USA; 2 The Grossman Institute for Neuroscience, University of Chicago, Chicago, IL, USA

## Abstract

Terminal selector-type transcription factors are key regulators of neuronal identity and function (Hobert and Kratsios, 2019; Kratsios and Hobert, 2018). Mechanistically, terminal selectors are thought to act directly through binding at the *cis*-regulatory region of genes (termed “terminal identity genes”) that encode, among others, neurotransmitter [NT] synthesis proteins, ion channels, neuropeptides, and cell adhesion molecules (Hobert and Kratsios, 2019; Kratsios and Hobert, 2018). Although dozens of terminal selectors have been described thus far for individual neuron types of the nematode *C. elegans *(Hobert, 2016), the identification of their target genes has primarily relied on candidate approaches and availability of markers for neuronal terminal identity. Hence, unbiased methods are needed to identify the full spectrum of terminal selector target genes in individual neuron types. This study focuses on the phylogenetically conserved terminal selector UNC-3/Ebf (member of the Collier/Olf/Ebf family), which controls cholinergic motor neuron (MN) identity in the ventral nerve cord of the nematode *C. elegans*. To identify novel UNC-3 target genes, we took advantage of the genome-wide binding map of UNC-3 from our previous Chromatin Immunoprecipitation followed by Sequencing (ChIP-Seq) analysis (Li et al., 2020). We generated transgenic reporter lines for ten putative terminal identity genes (*pxd-1, cal-2, lgc-4, ldb-1, nep-21, D2007.2, dmsr-2, ncs-2, npr-29, drn-1*)*, *whose expression patterns were largely unknown in *C. elegans.* Six of these reporter lines showed expression in ventral nerve cord MNs (*nep-21, D2007.2, dmsr-2, ncs-2, npr-29, drn-1), *whereas the remaining four* (pxd-1, cal-2, lgc-4, ldb-1) *showed expression in head and tail neurons, as well as some non-neuronal cells. Importantly, the number of ventral nerve cord MNs showing expression of the *nep-21, D2007.2, *and *dmsr-2 *reporters was significantly reduced in *unc-3* null mutant animals, thereby expanding the repertoire of known UNC-3 target genes in these cells. Altogether, this study demonstrates that transgenic reporter analysis guided by ChIP-Seq results is a relatively efficient approach for the identification and validation of transcription factor target genes.

**Figure 1. Survey for novel target genes of the terminal selector UNC-3:**
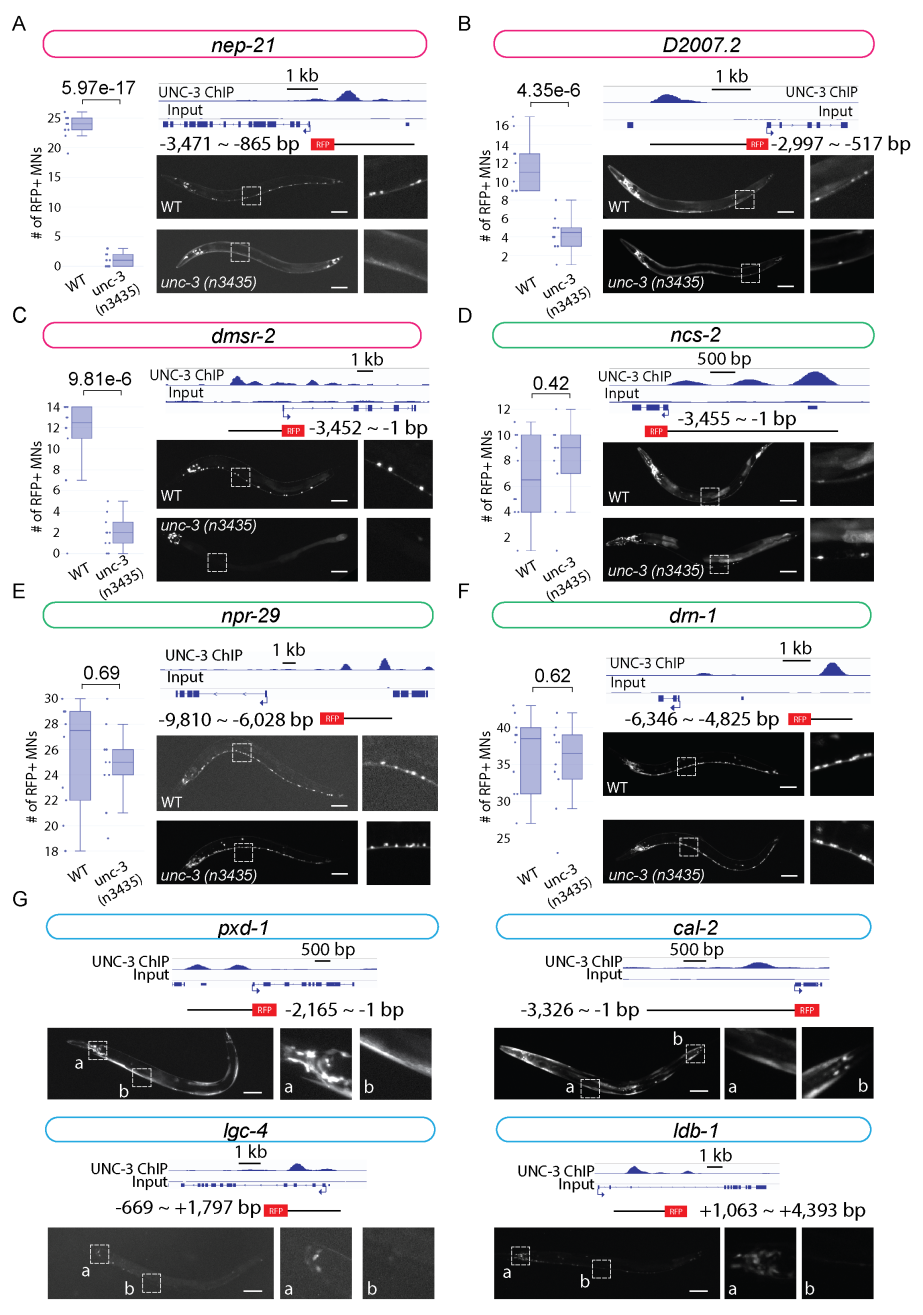
UNC-3 ChIP-Seq signal is aligned to specific gene loci of interest. Reporter constructs carrying the genomic region bound by UNC-3 followed by tagRFP are used to build transgenic animals, and the expression images are shown (scale bar: 50 μm). **A-F:** Six reporters are expressed in MNs and their *unc-3* dependency is assessed. The expression of fluorescent reporters is imaged and compared between wildtype and *unc-3 (n3435)* mutant animals. The number of ventral nerve cord MNs showing tagRFP expression is quantified for each genotype (N=10) and shown in boxplots. Student t test is performed and statistical significance is determined by p-value. Three reporter genes (*nep-21, D2007.2, dmsr-2*) show decrease of expression in *unc-3 (n3435)* mutants (**A-C**), while the remaining three genes (*ncs-2, npr-29, drn-1*) do not show changes in their expression (**D-F**). **G**: Four reporter genes (*pxd-1, cal-2, lgc-4,* and *ldb-1*) are not expressed in MNs of the ventral cord but are expressed in head neurons (all 4 genes), tail neurons (*pxd-1* and *cal-2*), and muscle or epidermal cells (*pxd-1* and *cal-2*). Zoomed insets are shown on the right of each image.

## Description

The role of UNC-3 in establishing and maintaining the cell identity of cholinergic motor neurons (MNs) has been investigated in several studies (Feng *et al.*, 2020; Kratsios *et al.*, 2011; Li *et al.*, 2020). Through a candidate approach, more than 50 terminal identity genes have been identified genetically as downstream targets of UNC-3. These genes encode proteins essential for the proper functions of cholinergic MNs and include acetylcholine biosynthesis components, ion channels, neurotransmitter receptors, and neuropeptides (Kratsios *et al.*, 2011). To test whether UNC-3 directly controls the expression of these genes, we recently performed Chromatin Immunoprecipitation followed by Sequencing (ChIP-Seq) analysis for UNC-3. Indeed, UNC-3 directly binds to the *cis*-regulatory region of these ~50 known target genes via a consensus DNA binding site, termed COE motif (Li *et al.*, 2020). This suggests that activating terminal identity genes through physical binding to their loci is a key strategy employed by UNC-3 to regulate cholinergic MN identity.

Here, we sought to expand the list of UNC-3 targets by taking advantage of the genome-wide binding map of UNC-3 from our previous ChIP-Seq analysis (Li *et al.*, 2020). We focused on testing 10 putative terminal identity genes (*pxd-1, cal-2, lgc-4, ldb-1, nep-21, D2007.2, dmsr-2, ncs-2, npr-29, drn-1*) that showed strong UNC-3 binding signal in their *cis-*regulatory region (enhancers, promoters or introns). To test the hypothesis that UNC-3 binding is required for gene expression, we first generated transgenic reporter lines for these 10 genes by amplifying the genomic region bound by UNC-3 and fusing it with a fluorescent reporter tagRFP. We found 6 reporter lines (*nep-21, D2007.2, dmsr-2, ncs-2, npr-29, drn-1*) with strong expression in ventral nerve cord MNs. Intriguingly, all these six genes have been independently validated by the CeNGEN consortium (Taylor *et al.*, 2021); RNA-Sequencing analysis shows high expression levels of *nep-21, D2007.2, dmsr-2, ncs-2, npr-29,* and *drn-1* in ventral cord MNs. To assess whether their expression in MNs is dependent on UNC-3, we crossed these reporter lines with *unc-3(n3435)* null mutants (Prasad *et al.*, 2008).

This analysis revealed three novel target genes of UNC-3 in cholinergic MNs: (a) *nep-21* is a member of the neutral endopeptidase family of zinc-metalloproteinases, which in mammals control hydrolysis of neuropeptides (Turner *et al.*, 2001), (b) *dmsr-2* is predicted to encode a protein that enables G protein-coupled peptide receptor activity (www.wormbase.org, WS281), and (c) *D2007.2* is an uncharacterized gene encoding a protein with an Immunoglobulin-like domain (www.wormbase.org, WS281). The number of cholinergic MNs showing expression of *nep-21::RFP*, *dmsr-2::RFP* and *D2007.2::RFP* is significantly decreased in *unc-3(n3435)* animals (Figure 1A-C), suggesting UNC-3 acts as an activator for these genes. Since our reporter constructs carry the UNC-3 binding region (putative enhancer region), these data strongly suggest that UNC-3 regulates the expression of *nep-21, D2007.2,* and *dmsr-2* by direct binding to their gene loci and activating the transcriptional machinery. On the other hand, we did not detect statistically significant differences in the expression of *ncs-2*, *npr-29,* and *drn-1* in cholinergic MNs of *unc-3(n3435)* when compared to wild-type animals (Figure 1D-F). This does not necessarily exclude UNC-3 as a regulator of these genes. Regulation of gene transcription is a complex process, which commonly involves more than one regulator. Such gene regulators may co-regulate the expression of the same gene while acting redundantly, which can be the case for *ncs-2*, *npr-29,* and *drn-1*. Nevertheless, these findings suggest that transgenic reporter analysis guided by ChIP-Seq results is a relatively efficient approach for the identification and validation of transcription factor target genes.

We also found that RFP reporter lines for the remaining four genes *(pxd-1, cal-2, lgc-4, ldb-1)* do not show expression in ventral nerve cord MNs, despite UNC-3 binding on their *cis-*regulatory regions (Figure 1G). Since the genomic regions in our reporter lines are selected mainly based on UNC-3 binding signal from ChIP-Seq data, it is possible they contain partial regulatory elements necessary for gene expression in MNs, and therefore may not report the endogenous expression pattern of these genes. Moreover, we found that these RFP reporter lines are expressed in head neurons, tail neurons, and muscle cells (Figure 1G). UNC-3 is known to be expressed in a small group of head (AVA, AVB, AVD, AVE) and tail (PDA, PDB, PVC, DVA, PVN) neurons (Pereira *et al.*, 2015). Future studies are needed to determine whether *pxd-1, cal-2, lgc-4,* and *ldb-1* are expressed in any of these *unc-3-*expressing neurons. Lastly, our observations suggest that transgenic reporter analysis guided by ChIP-Seq can be used for the generation of tissue and neuron-specific reporter lines.

Altogether, we generated transgenic reporter lines for ten putative terminal identity genes in *C. elegans,* whose expression patterns were largely unknown. These tagRFP reporter lines will be useful tools for researchers who intend to investigate further the functions of these genes in the *C. elegans* nervous system. Lastly, our reporter analysis of ChIP-Seq-defined enhancer regions identified three novel target genes (*nep-21, dmsr-2, D2007.2*) of the terminal selector UNC-3 in cholinergic MNs, expanding the repertoire of its target genes.

## Methods

**Generation of transgenic animals carrying transcriptional fusion reporters**

Reporter gene fusions for *cis*-regulatory analyses and validation of newly identified UNC-3 target genes were made with PCR fusion (Hobert *et al.*, 2002). Genomic regions were amplified and fused to the coding sequence of tagrfp followed by the *unc-54* 3’ UTR. PCR fusion DNA fragments were injected into young adult *pha-1(e2123)* hermaphrodites at 50 ng/µl together with *pha-1* (pBX plasmid) as co-injection marker (50 ng/µl).

## Reagents

**Table:** 

Strain code	Gene	Description	Availability at CGC
KRA582	*pxd-1*	*pha-1 (e2123); kasEx271 [pxd-1::RFP+pha-1 cDNA]* RFP fused with genomic region (-2,165 ~ -1 bp)	Yes
KRA583	*pxd-1*	*pha-1 (e2123); kasEx272 [pxd-1::RFP+pha-1 cDNA]* RFP fused with genomic region (-2,165 ~ -1 bp)	Yes
KRA584	*cal-2*	*pha-1 (e2123); kasEx273 [cal-2::RFP+pha-1 cDNA]* RFP fused with genomic region (-3,326 ~ -1 bp)	Yes
KRA585	*cal-2*	*pha-1 (e2123); kasEx274 [cal-2::RFP+pha-1 cDNA]* RFP fused with genomic region (-3,326 ~ -1 bp)	Yes
KRA586	*lgc-4*	*pha-1 (e2123); kasEx275 [lgc-4::RFP+pha-1 cDNA]* RFP fused with genomic region (-669 ~ +1,797 bp)	Yes
KRA587	*lgc-4*	*pha-1 (e2123); kasEx276 [lgc-4::RFP+pha-1 cDNA]* RFP fused with genomic region (-669 ~ +1,797 bp)	Yes
KRA588	*ldb-1*	*pha-1 (e2123); kasEx277 [ldb-1::RFP+pha-1 cDNA]* RFP fused with genomic region (+1,063 ~ +4,393 bp)	Yes
KRA589	*ldb-1*	*pha-1 (e2123); kasEx278 [ldb-1::RFP+pha-1 cDNA]* RFP fused with genomic region (+1,063 ~ +4,393 bp)	Yes
KRA590	*nep-21*	*pha-1 (e2123); kasEx279 [nep-21::RFP+pha-1 cDNA]* RFP fused with genomic region (-3,471 ~ -865 bp)	Yes
KRA591	*nep-21*	*pha-1 (e2123); kasEx280 [nep-21::RFP+pha-1 cDNA]* RFP fused with genomic region (-3,471 ~ -865 bp)	Yes
KRA592	*D2007.2*	*pha-1 (e2123); kasEx281 [D2007.2::RFP+pha-1 cDNA]* RFP fused with genomic region (-2,997 ~ -517 bp)	Yes
KRA593	*D2007.2*	*pha-1 (e2123); kasEx282 [D2007.2::RFP+pha-1 cDNA]* RFP fused with genomic region (-2,997 ~ -517 bp)	Yes
KRA594	*dmsr-2*	*pha-1 (e2123); kasEx283 [dmsr-2::RFP+pha-1 cDNA]* RFP fused with genomic region (-3,452 ~ -1 bp)	Yes
KRA595	*ncs-2*	*pha-1 (e2123); kasEx284 [ncs-2::RFP+pha-1 cDNA]* RFP fused with genomic region (-3,455 ~ -1 bp)	Yes
KRA596	*ncs-2*	*pha-1 (e2123); kasEx285 [ncs-2::RFP+pha-1 cDNA]* RFP fused with genomic region (-3,455 ~ -1 bp)	Yes
KRA597	*npr-29*	*pha-1 (e2123); kasEx286 [npr-29::RFP+pha-1 cDNA]* RFP fused with genomic region (-9,810 ~ -6,028 bp)	Yes
KRA598	*npr-29*	*pha-1 (e2123); kasEx287 [npr-29::RFP+pha-1 cDNA]* RFP fused with genomic region (-9,810 ~ -6,028 bp)	Yes
KRA599	*drn-1*	*pha-1 (e2123); kasEx288 [drn-1::RFP+pha-1 cDNA]* RFP fused with genomic region (-6,346 ~ -4,825 bp)	Yes
KRA600	*drn-1*	*pha-1 (e2123); kasEx289 [drn-1::RFP+pha-1 cDNA]* RFP fused with genomic region (-6,346 ~ -4,825 bp)	Yes
MT10785	*unc-3*	*unc-3(n3435)* X	No
KRA644	*unc-3*	*unc-3(n3435); kasEx289[drn-1::RFP+pha-1 cDNA]*	No
KRA645	*unc-3*	*unc-3(n3435); kasEx281[D2007.2::RFP+pha-1 cDNA]*	No
KRA646	*unc-3*	*unc-3(n3435); kasEx286[npr-29::RFP+pha-1 cDNA]*	No
KRA647	*unc-3*	*unc-3(n3435); kasEx279[nep-21::RFP+pha-1 cDNA]*	No
KRA648	*unc-3*	*unc-3(n3435); kasEx284[ncs-2::RFP+pha-1 cDNA]*	No
